# Photosynthetic Response Mechanism of Soil Salinity-Induced Cross-Tolerance to Subsequent Drought Stress in Tomato Plants

**DOI:** 10.3390/plants9030363

**Published:** 2020-03-16

**Authors:** Xiaolong Yang, Yangyang Li, Hangbing Chen, Juan Huang, Yumeng Zhang, Mingfang Qi, Yufeng Liu, Tianlai Li

**Affiliations:** 1 Horticulture Department, Shenyang Agricultural University, No. 120 Dongling Road, Shenhe District 110866, China; 20161078@stu.syau.edu.cn (X.Y.);; 2 Key Laboratory of Protected Horticulture of Ministry of Education, No. 120 Dongling Road, Shenhe District 110866, China; 3 National & Local Joint Engineering Research Center of Northern Horticultural Facilities Design & Application Technology (Liaoning), No. 120 Dongling Road, Shenhe District 110866, China

**Keywords:** soil salinity, cross-tolerance, drought, photosynthetic acclimation, ATP synthase, proton motive force

## Abstract

Soil salinization and water shortage cause ion imbalance and hyperosmoticity in plant cells, adversely impairing photosynthesis efficiency. How soil salinity-induced photosynthetic acclimation influences the cross-tolerance to drought conditions represents a promising research topic. This study was to reveal the photosynthetic mechanism of soil salinity-induced resistance to the subsequent drought stress in tomato leaves through comprehensive photosynthesis-related spectroscopy analysis. We conducted soil salinity pretreatment and subsequent drought stress experiments, including irrigation with 100 mL water, 100 mL 100 mM NaCl solution (NaCl100), 50 mL water, and 50 mL 100 mM NaCl solution (NaCl50) for five days, followed by five-day drought stress. The results showed that soil salinity treatment by NaCl decreased the rate of photosynthetic gas exchange but enhanced CO_2_ assimilation, along with photosystem II [PS(II)] and photosystem I [PS(I)] activity and photochemical efficiency in tomato plants compared with water pretreatment after subsequent drought stress. NaCl100 and NaCl50 had the capacity to maintain non-photochemical quenching (NPQ) of chlorophyll fluorescence and the cyclic electron (CEF) flow around PSI in tomato leaves after being subjected to subsequent drought stress, thus avoiding the decrease of photosynthetic efficiency under drought conditions. NaCl100 and NaCl50 pretreatment induced a higher proton motive force (pmf) and also alleviated the damage to the thylakoid membrane and adenosine triphosphate (ATP) synthase of tomato leaves caused by subsequent drought stress. Overall, soil salinity treatment could enhance drought resistance in tomato plants by inducing NPQ, maintaining CEF and pmf that tradeoff between photoprotection and photochemistry reactions. This study also provides a photosynthetic perspective for salt and drought cross-tolerance.

## 1. Introduction

Among adverse abiotic stressors, soil salinization and water shortage constitute long-term challenges for growers and scientists. A large area of global land is subjected to soil salinization during the process of field crop production or intensive vegetable production. Soil salinization is determined by the combined effects of certain natural conditions and improper farming practices, especially the extensive and unscientific farming operation, incomplete irrigation system, and inadequate management. With global warming, the problem of soil salinization will become even more severe in low and mid-latitude regions [1]. Soil salinity stress alters the water status of plant cells, disturbing initial growth and causing ion imbalance or toxicity consequent to the excessive uptake of ions, which further adversely affects the growth, development, and photosynthesis efficiency [2]. Ion imbalance and hyperosmoticity in plant cells represent the primary effects, which are typically accompanied by oxidative damage resulting from the burst of reactive oxygen species (ROS) [3].

Soil salinity-induced osmotic tolerance occurs relatively quickly to alter the leaf anatomy, causing rapid stomatal closure to preserve water and protect the photosynthetic apparatus. This is followed by a slow phase pertaining to the build-up of cytotoxic ion levels, which causes physiological damage. Plants must alleviate the damage, re-establish homeostatic conditions in response to soil salinity through multiple adaptive mechanisms [4]. The increase in cytosolic free calcium concentrations ([Ca^2+^]_cyt_) induced by soil salinity activates the salt overly sensitive (SOS) signaling pathway, which further extrudes sodium ions into the apoplast to maintain ionic homeostasis [5]. Mitogen activated protein kinase (MAPK) cascades mediate ROS signaling, with sucrose nonfermenting 1-related protein kinase 2 (SnRK2) proteins being involved in the regulation of stress-related gene expression to maintain osmotic homeostasis under salt stress [6].

Photosynthesis is sensitive to soil salinization and water deficit owing to rapid stomatal closure and interference with photosynthetic electron transport; it is generally considered that the energy produced by photosynthesis is redistributed from growth into stress defense [7,8]. Decrease in photochemical reaction efficiency results in insufficient production of the reduction force of adenosine triphosphate (ATP) and nicotinamide adenine dinucleotide phosphate (NADPH), which reduces the capacity of carbon dioxide assimilation. Alternatively, leaf photosynthesis adapts to salt stress through a variety of photoprotection pathways to avoid oxidative damage to the photosynthetic apparatus caused by the excessive accumulation of ROS [9,10]. In particular, fast induction and relaxation of non-photochemical quenching of chlorophyll fluorescence (NPQ) plays a key role in protecting plants against photoinhibition [11].

Plants are often subjected to multiple abiotic stresses simultaneously, affecting their growth and development. Plant exposure to a non-lethal condition can increase its ability to resist other adverse environmental, this phenomenon is termed plant cross-tolerance [12,13]. Jiang et al. (2017) reported that tomato plants in soil pre-treated with salt were able to retain higher leaf mass per area, higher total Chl and Car contents, and higher photosynthetic activities than those in fumigated plants not pre-treated [14]. Mild cold, paraquat, and drought pretreatment can also induce tolerance to subsequent chilling, photooxidative agents, and drought stresses in tomato plants, the production of *RBOH1*-dependent H_2_O_2_ and subsequent activation of MPK1/2 potentially being involved in this acclimation-induced cross-tolerance [13]. In addition, drought priming-induced resistance to heat in tall fescue and *Arabidopsis*, along with heat-shock and NaCl-induced resistance to ultraviolet-B (UV-B) in barley, has also been recently reported [15,16].

In China, tomato is the most important vegetable in greenhouse production. Extensive fertilization, inappropriate irrigation, and years of continuous cropping have aggravated the soil salinization and severely restricted tomato production. Soil salinity is commonly accompanied with the occurrence of drought conditions during plant growth; photosynthesis is the most vulnerable target due to its operation being highly dependent on environmental conditions. Even though numerous studies have focused on the response of plant photosynthesis to single stress factors, the role and response mechanism of photosynthetic acclimation in cross-tolerance is not clear. Enhancing photosynthetic acclimation is a promising strategy to increase plant cross-tolerance [17,18]. To achieve this, it is necessary to understand how soil salinity influences photosynthesis by tradeoff between photoprotection and photochemistry and whether the photosynthetic acclimation can enhance cross tolerance to other stressors such as drought. In this study, we aimed to elucidate the photosynthetic response mechanism of soil salinity-induced resistance to the subsequent drought stress in tomato leaves.

## 2. Results 

### 2.1. Gas Exchange Parameters under Soil Salinity and Subsequent Drought Stress

**Figure 1:**
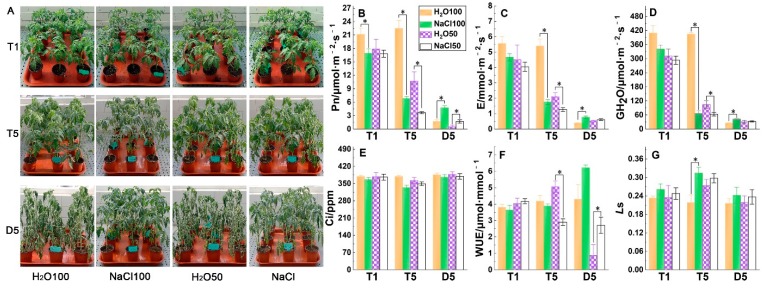
The effect of soil salinity on plant phenotype and photosynthetic gas exchange parameters of tomato leaves under subsequent drought stress. Tomato plants growth state under different treatments at different stages (**A**), the effect of soil salinity on net CO_2_ assimilation rate (**B**), transpiration rate (**C**), stomatal conductance (**D**), intercellular CO_2_ concentration (**E**), water use efficiency (WUE) (**F**), and stomatal limitation value (Ls) (**G**) in tomato leaves under subsequent drought stress. T1, the first day following soil salt pre-treatment; T5, the fifth day following soil salt pre-treatment; D5, the fifth day after the subsequent drought treatment. The results are displayed as mean values of six independent biological replicates, means ± standard deviation (SD), *P < 0.05, student’s t-test.

Plant growth under NaCl100 and NaCl50 treatment exhibited withering to some extent on T5 (the fifth day following soil salt pre-treatment), as did H_2_O50 in comparison with H_2_O100. Conversely, the plants irrigated with water showed rapid and obviously more severe wilting than salt-treated plants after subsequent drought treatment for five days (Figure 1A). The net CO_2_ assimilation rate, Pn, of tomatoes under NaCl100 and NaCl50 was significantly lower than that of H_2_O100 on T1 (the first day following soil salt pre-treatment), indicating that the CO_2_ assimilation rate of tomato leaves is sensitive to salt treatment. The decline had further widened by the T5 stage, with the Pn of NaCl100 treatment being significantly lower than that of H_2_O100 and the NaCl50 Pn significantly lower than that of H_2_O50. Pn of NaCl100 and NaCl50 tomato plants showed a much smaller decrease than the water controls when plants were subjected to subsequent drought stress for five days (Figure 1B). The response of E and GH_2_O of tomatoes to the treatments exhibited a similar trend as Pn (Figure 1C,D). The Ci of NaCl100 was significantly lower than that of H_2_O100 on T5, whereas no difference was observed on D5 (the fifth day after the subsequent drought treatment), which may be due to the severe loss of water or the change of leaf morphology (Figure 1E). The water use efficiency (WUE) of NaCl50 was significantly lower than that of H_2_O50, whereas the NaCl100 and NaCl50 WUE was significantly higher than that of H_2_O100 and H_2_O50, respectively, when plants were subjected to subsequent drought stress for five days (Figure 1F). The *Ls* of tomatoes under NaCl100 was significantly higher than that of H_2_O100 on T5, but there was no significant difference between treatments on D5 (Figure 1G). These results suggested that soil salinity conferred by either 100 or 50 mM NaCl could decrease the rate of photosynthesis gas exchange but enhance CO_2_ assimilation when the plants suffered subsequent drought stress.

### 2.2. PSI and PSII Activity under Soil Salinity and Subsequent Drought Stress

**Figure 2:**
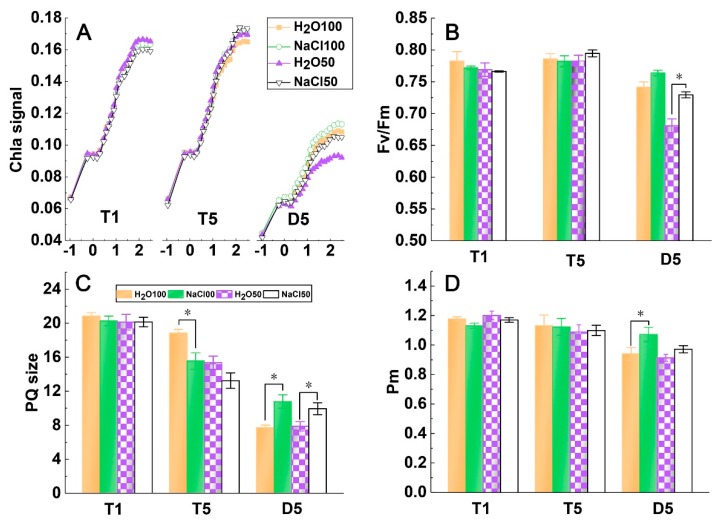
The effect of soil salinity on the photosynthetic activity of PSI and PSII of tomato leaves under subsequent drought stress. The fast induction curve of chlorophyll a fluorescence (**A**), the maximal quantum efficiency of PSII (Fv/Fm) (**B**), plastoquinone (PQ) size (**C**) and the maximal redox state of PSI (Pm) (**D**) of tomato leaves under subsequent drought stress. The pattern in (**A**) represents the redox kinetic curve of P700. T1, the first day following soil salt pre-treatment; T5, the fifth day following soil salt pre-treatment; D5, the fifth day after the subsequent drought treatment. The results are displayed as mean values of three independent biological replicates, means ± SD, * *P* < 0.05, student’s t-test.

The chlorophyll fluorescence transient was detected to analyze the PSII activity of tomato leaves. All fast induction curves of chlorophyll a fluorescence showed a typical polyphasic rise of O-J-I-P (Figure 2A). The results indicated that the chlorophyll fluorescence transients did not obviously differ in T1 and T5, whereas they obviously decreased after the five-day drought treatment, suggesting that the drought treatment aggravated PSII photoinhibition. In addition, the difference between treatments in D5 may reflect that soil salinity could enhance the activity of PSII when plants were subjected to subsequent drought stress (Figure 2A). Fv/Fm of the tomato plants from different treatments in T1 and T5 did not significantly differ, whereas Fv/Fm of the NaCl100 and NaCl50 treatment was significantly higher than that of H_2_O100 and H_2_O50, respectively, when plants suffered subsequent drought stress for five days (Figure 2B). The calculated plastoquinone (PQ) sizes of NaCl100, H_2_O50, and NaCl50 were significantly lower than those of H_2_O100 at T5, suggesting that soil salinity and mild drought decreased the size of the electron carrier. In addition, the results revealed significantly higher PQ size values under NaCl100 and NaCl50 treatment relative to those of H_2_O100 and H_2_O50, respectively, at D5, indicating that soil salinity pretreatment could enhance the electron transfer capacity of PQ under subsequent drought stress (Figure 2C). As the Pm can reflect the activity of PS1 to some extent, the lack of significant difference in Pm between treatments at T1 and T5 illustrated a decreasing trend of H_2_O100, H_2_O50, and NaCl50 after subsequent drought stress, whereas the NaCl100 treatment exhibited a significantly higher Pm value than that of the other treatments at D5 (Figure 2D). These results indicated that the soil salinity treatment could enhance the photochemical efficiency of PSII and increase the PQ size when plants were subjected to subsequent drought stress, whereas the activity of PSI exhibited a relative strong capacity to avoid damage by salt and drought stress, as evidenced by the lack of significant difference between treatments.

### 2.3. PSI and PSII Energy Conversion under Soil Salinity and Subsequent Drought Stress

**Figure 3:**
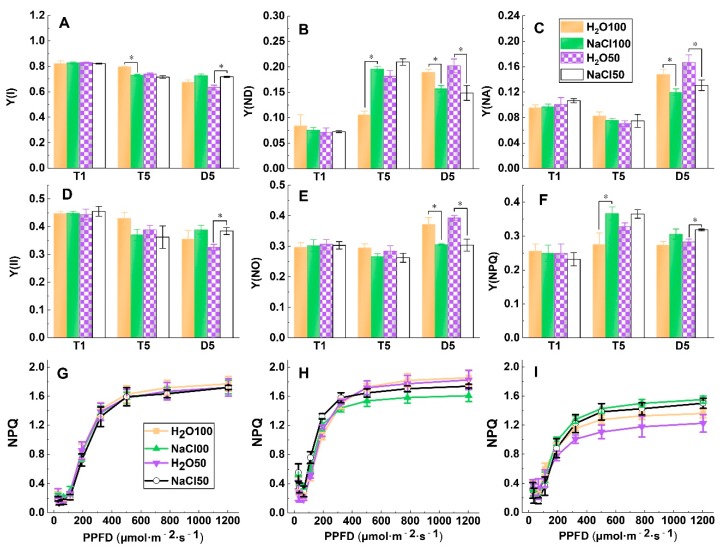
The effect of soil salinity on energy conversion in PSI and PSII of tomato leaves under subsequent drought stress. Y(I) (**A**); Y(ND) (**B**); Y(NA) (**C**); Y(II) (**D**); Y(NO) (**E**); Y(NPQ) (**F**); and the rapid light response curves of NPQ of tomato plants at T1 (**G**); T5 (**H**); and D5 (**I**). T1, the first day following soil salt pre-treatment; T5, the fifth day following soil salt pre-treatment; D5, the fifth day after the subsequent drought treatment. The results are displayed as mean values of three independent biological replicates, means ± SD, * *P* < 0.05, student’s t-test.

The Y(II) and Y(I) were significantly decreased under conditions of soil salinity and moderate drought stress compared to those of normally watered plants at T5. In comparison, the values of Y(II) and Y(I) were higher under soil salinity pretreatment compared to those with water treatment at D5, reaching a significant level between H_2_O50 and NaCl50 (Figure 3A,D). The Y(ND) was significantly increased under NaCl100 and NaCl50 treatment compared to those under H_2_O100 and H_2_O50 treatment at T5, respectively. Y(ND) of NaCl100 and NaCl50 plants both decreased and showed significantly lower values than water controls at D5 (Figure 3B). The Y(NA) show no significantly difference between treatments at T1 and T5; however, although the values of all treatments increased after subsequent drought stress for five days, soil salinity pretreatments showed significantly lower Y(NA) compared with that of the water pretreatments (Figure 3C). The Y(NO) did not significantly differ between treatments at T1 and T5, whereas that of NaCl100 and NaCl50 was significantly higher than the Y(NO) of H_2_O100 and H_2_O50 at D5, respectively (Figure 3E). The Y(NPQ) of plants under NaCl100 and NaCl50 was significantly higher than that of H_2_O100 and H_2_O50 at T5, respectively, and exhibited a similar trend at D5 (Figure 3F). We further analyzed the RLCs of NPQ; because this parameter is sensitive to excess absorption of light energy, the NPQ increases rapidly with increasing light intensity and tends to stabilize when light intensity reaches 400 μmol·m^−2^·s^−1^ (Figure 3G–I). No difference was observed between treatments from low to high light intensities at T1 (Figure 3G). NPQs of tomato leaves under both treatments reached a steady state when light intensity reached 400 μmol·m^−2^·s^−1^, and the values of the NaCl100 at steady state were significantly lower compared with H_2_O100 at T5 (Figure 3H). In contrast, upon subjection to subsequent five days of drought stress, the NPQ of tomato leaves under NaCl100 was significantly higher than that under H_2_O100, similar results were obtained when comparing NaCl50 with H_2_O50 (Figure 3I). These results indicated that the soil salinity pretreatment could affect PSI and PSII energy conversion to alleviate photosynthetic electron transport chain of tomato plants from the damages induced by subsequent drought stress.

### 2.4. Liner and Cyclic Photosynthetic Electron Transport under Soil Salinity and Subsequent Drought Stress

**Figure 4:**
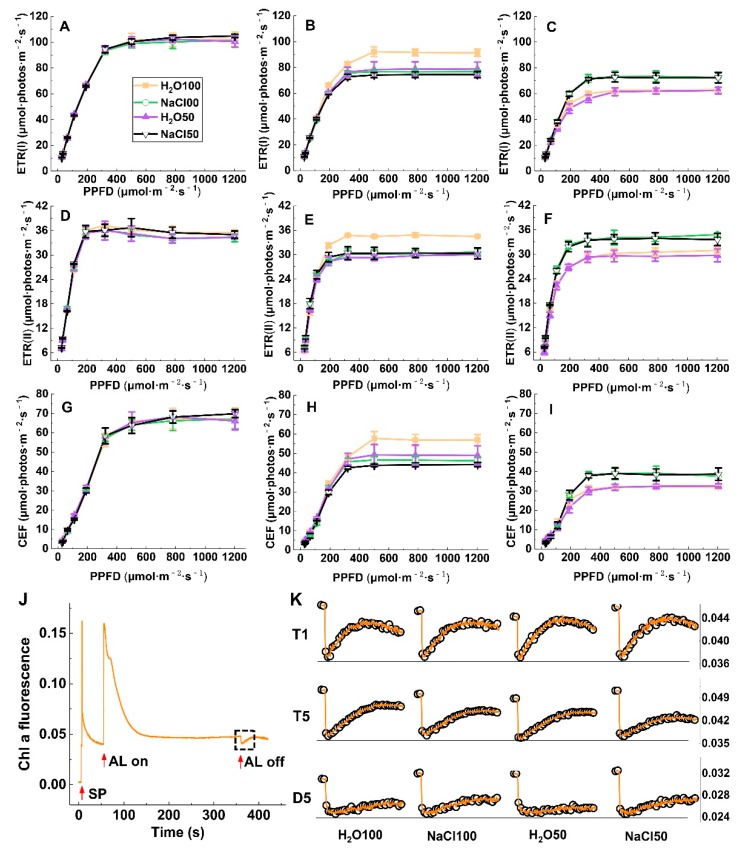
The effect of soil salinity on linear and cyclic photosynthetic electron transport of tomato leaves under subsequent drought stress. A-C, ETR(I) at T1 (**A**); T5 (**B**); and D5 (**C**). D-G, ETR (II) at T1 (**D**); T5 (**E**); and D5 (**F**). G-I, CEF at T1 (**G**); T5 (**H**); D5 (**I**); A standard measurement procedure to record the transient increase in chlorophyll fluorescence after switching off actinic light (**J**). Transient increase in chlorophyll fluorescence signal kinetics curve of dark-adapted tomato leaves at T1, T5, and D5 (**K**). T1, the first day following soil salt pre-treatment; T5, the fifth day following soil salt pre-treatment; D5, the fifth day after the subsequent drought treatment. The results are displayed as mean values of three independent biological replicates, means ± SD, * *P* < 0.05, student’s t-test.

ETR(II), ETR(I), and CEF in tomato leaves rapidly increased with increasing light intensity. The ETRII increase slowed and became steady after the light intensity reached 200 μmol·m^−2^·s^−1^ and that of ETR(I) and CEF slowed and became steady after 400 μmol·m^−2^·s^−1^ light intensity, although no significant difference of these parameters was observed between treatments at T1 (Figure 4A,D,G). In comparison, the ETR(I), ETR(II), and CEF values of tomato leaves under NaCl100, H_2_O50, and NaCl50 were significantly lower compared with those under H_2_O100 at a steady state at T5 (Figure 4B,E,H), indicating that soil salinity and moderate water deficit could reduce the rate of both linear and cyclic electron transport. When plants were subjected to subsequent five-day drought stress, ETR(II), ETR(I), and CEF in tomato leaves under NaCl100 and NaCl50 were significantly higher than those under H_2_O100 and H_2_O50, respectively, when reaching the steady state (Figure 4C,F,I). The post-illumination-induced transient chlorophyll fluorescence increase was measured to estimate CEF (Figure 4J). All treatments at T1 showed a clear post-illumination-induced transient chlorophyll fluorescence increase to some extent with attenuation of NaCl100, H_2_O50, and NaCl50 compared with the H_2_O100 treatment at T5. After the subsequent five-day drought stress, the post-illumination-induced transient chlorophyll fluorescence increase weaken further, whereas the increase in NaCl100 and NaCl50 leaves was markedly increased compared with that of the H_2_O100 and H_2_O50 treatment (Figure 4K). These results demonstrated that the soil salinity pretreatment likely plays an important role in maintaining CEF.

### 2.5. Response of pmf and ATP-synthase Activity to Soil Salinity and Subsequent Drought Stress

**Figure 5:**
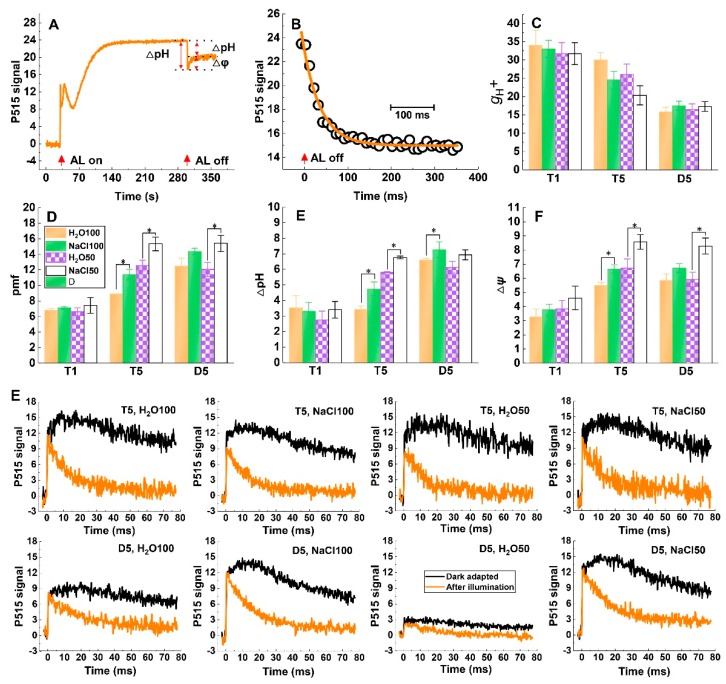
The effect of soil salinity on proton motive force (pmf), thylakoid membrane integrity, and ATP synthase activity of tomato leaves under subsequent drought stress. Typical slow dark–light–dark-induced P515 signal kinetics curve of dark-adapted tomato leaves (**A**); Rapid relaxation of the electrochromic shift (ECS) signal after the actinic light was turned off, and gH+ was estimated as the inverse of the time constant of the first-order ECS relaxation (**B**); gH+ (**C**); Pmf (**D**); ΔpH (**E**); Δψ (**F**); The changes of ECS signal induced by single turnover flash (ST, 50 ms) after 1 h of dark adaptation and illuminated at 630 μmol·photons·m^−2^·s^−1^ for 6 min followed by 4 min dark adaptation (**G**). T1, the first day following soil salt pre-treatment; T5, the fifth day following soil salt pre-treatment; D5, the fifth day after the subsequent drought treatment. The results are displayed as mean values of three independent biological replicates, means ± SD, * *P* < 0.05, student’s t-test.

The formation of the pmf, which drives proton efflux across the thylakoid membrane through the chloroplastic ATP synthase, and its components Δψ and ΔpH in leaves can be monitored in vivo after dark adaptation for 1 h by analyzing the light-off responses of the electrochromic shift (ECS) signal (Figure 5A). The inverse of the time constant of the first-order ECS relaxation was considered to be *g*H^+^, which reflects the chloroplastic ATP synthase activity (Figure 5B). Our results showed that the *g*H^+^ values of NaCl100 and NaCl50 treatment were lower than that of H_2_O100 H_2_O50 treatment at T5, respectively, whereas the *g*H^+^ values both decreased and showed no difference between treatments after the subsequent five-day drought stress, respectively (Figure 5C). This indicated that soil salinity could reduce the activity of chloroplastic ATP synthase but maintain higher ATP synthase activity when the samples were subjected to subsequent drought stress. The results of pmf, ΔpH, and Δψ showed similar trends during the experiment, with soil salinity stress increasing these parameters at T5. Notably, the soil salinity pretreatment enhanced pmf, ΔpH, and Δψ compared to those of the water treatment after the subsequent five-day drought stress (Figure 5D–F). The slow decay of the ECS signal after dark-adaptation reflects high membrane integrity and the fast decay after illumination reflects high ATP-synthase activity [19,20]. The kinetic curve exhibited no obvious difference between treatments at T5; however, the slow decay of the ECS signal after dark-adaptation and the fast decay after illumination of the NaCl100- and NaCl50-treated plants were obviously higher compared with those of H_2_O100 and H_2_O50, respectively (Figure 5G). This implied that the integrity of the thylakoid membrane was impaired and that ATP-synthase activity was affected more compared to the plants treated with NaCl100 and NaCl50. As a result, the rate of lumen-to-stroma proton transfer via ATP-synthase was decreased, which increase the formation of NPQ when plants were subjected to subsequent drought stress. Taken together, the results reveal pmf and ATP synthase involves in soil salinity-induced cross-tolerance to drought.

## 3. Discussion

**Figure 6:**
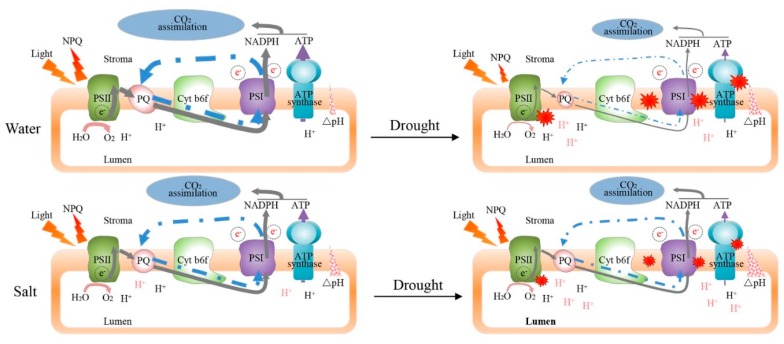
Schematic presentation of the photosynthetic response mechanism of soil salinity-induced cross-tolerance to drought stress in tomato plants. Soil salinity pretreatment can enhance CO_2_ assimilation efficiency and the activity of PSI and PSII compared with water controls when plants suffer subsequent drought stress. Soil salinity can induce PSI non-photochemical energy dissipation owing to the donor-side limitation and regulated energy dissipation in PSII, but can reduce PSI non-photochemical energy dissipation owing to both the donor and accept-side limitation. Moreover, soil salinity can maintain higher NPQ at PSII, thereby protecting PSI and PSII in tomato plants from the damage caused by subsequent drought stress. Soil salinity significantly reduces the rate of both linear and cyclic electron transport while alleviating the decrease of ETR(II), ETR(I), and CEF in tomato leaves exposed to subsequent drought stress; the maintenance of CEF may serve as an important acclamation mechanism to protect PSI from the damages caused by subsequent drought stress. The soil salinity treatment had the capacity to decrease the rate of lumen-to-stroma proton transfer via ATP-synthase, which induces a greater acidification in the thylakoid lumen so that it can induce photoprotection through NPQ upon plant exposure to subsequent drought stress.

In this study, we explored the photosynthetic response mechanism of soil salinity-induced resistance to subsequent drought stress in tomato leaves through a comprehensive photosynthesis-related spectroscopy analysis. As plants grown under field conditions are constantly exposed to environmental changes, crops inevitably endure more than one abiotic stress factor during production either simultaneously or sequentially. These adverse conditions, especially soil salinity and water deficit, are common in vegetable production. The adverse effects also serve as driving forces for improved plant resistance and adaptability, with acclimation-induced cross-tolerance allowing plant survival in the fluctuating environment. Our results revealed that cross-tolerance exists between soil salinity and the subsequent drought stress, and soil salinity pretreatment can alleviate drought-induced damage through photosynthesis acclimation (Figure 6).

The maintenance of photosynthetic efficiency constitutes an important mechanism of soil salinity-induced cross-tolerance. Our results showed that soil salinity could enhance CO_2_ assimilation and photochemical efficiency, and increase PQ size when plants suffer subsequent drought stress. We consider that this enhancement is related to the photoprotective mechanism induced by the soil salinity pretreatment. Similarly, a previous study found that the reduced leaf photosynthesis upon sulfur treatment was due to non-stomatal limitation, as the reduction of photochemical reactions causes more energy to be dissipated as heat [13]. NPQ represent the response of the photosynthetic membrane to excess light intensity, it is crucial for balancing the energy distribution between growth and stress response by regulating photoinhibition and photoprotection [11,21,22,23]. In the present study, the soil salinity pretreatment could induce PSI non-photochemical energy dissipation owing to the donor-side limitation and regulated energy dissipation in PSII, and maintained higher NPQ at PSII. The dissipation of excess energy during photochemical reaction in response to the soil salinity play an important role in protect photosynthetic apparatus from the subsequent drought stress damage.

Apart from the canonical linear electron flow from water to CO_2_, CEF contributes to the photoprotection of both PSI and PSII and ΔpH formation for supplying extra ATP, which could be used for sustaining photosynthesis and enhancing plant tolerance to fluctuating stress conditions [24]. In the present study, ETR(I), ETR(II), and CEF in tomato leaves under NaCl100 and NaCl50 were significantly lower compared with those of the H_2_O100 and H_2_O50 treatment at T5, respectively (Figure 4). Conversely, ETR(II), ETR(I), and CEF in tomato leaves were significantly higher for NaCl100 and NaCl50 than those for H_2_O100 and H_2_O50 treatments, respectively, when plants were subjected to subsequent five-day drought stress (Figure 4). The photoprotective role of CEF has been widely reported to participate in coping with high light, heat, and chilling stress [25]. CEF is required for both the donor-side and acceptor-side regulation and contribute to pmf formation, essential for PSI photoprotection [26,27]. Results of the present study clearly indicated that the soil salinity pretreatment is important for maintaining cyclic electron transfer around PSI. The higher CEF during photochemical reaction help to protect the activity of PSI and PSII from the subsequent drought stress damage through regulation the donor-side and acceptor-side of PSI energy dissipation.

Together, △pH and △ψ constitute pmf, which is measured by the signal change of ECS and drives ATP production via ATP synthase [28]. The decrease in pH within the thylakoid lumen is an immediate signal of excessive light that triggers the feedback regulation of light harvesting by NPQ [29]. Several previous studies have revealed the importance of chloroplastic ATP synthase for PSI and PSII photoprotection through suppressing ROS production in PSI and adjusting the redox state of reaction center chlorophyll in PSI during photosynthesis upon fluctuating conditions [28,30]. In the present study, soil salinity increased pmf, ΔpH, and Δψ at T5, and also enhanced both parameters compared to those of the water treatment following subsequent five-day drought stress (Figure 5). Moreover, the gH^+^ values of H_2_O100 and H_2_O50 were significantly higher than that of NaCl100and NaCl50 at T5, respectively. The gH^+^ of water controls decreased more rapidly than with soil salinity treatments, and there was no significant difference between treatments after the subsequent five-day drought stress, respectively (Figure 5C). The integrity of the thylakoid membrane was impaired and ATP-synthase activity was affected more compared to the plants treated with NaCl100 and NaCl50 after the subsequent five-day drought stress (Figure 5G). As the activity of chloroplastic ATP synthase determines gH^+^, its significant influence on the formation of pmf. The increased pmf induced by soil salinity pretreatment triggers NPQ, which may serve as an important photoprotection mechanism of soil salinity-induced cross-tolerance to subsequent drought stress. 

From what has been discussed above, we proposed a possible model that the inducing of NPQ and maintaining the cyclic electron flow and ATP synthase activity are crucial photosynthetic acclimation mechanisms in soil salinity-induced cross-tolerance to subsequent drought stress according to the observation results of this study (Figure 6). However, whether the changes of photosynthetic parameters are the consequences of salt stress or part of protective mechanisms induced by salt acclimation is unknown. Based on the results of this study, photoprotective mechanisms were activated in response to the soil salinity; the acquired photosynthetic acclimation can enhance the photochemical reaction efficiency in tomato plants under subsequent drought stress. In addition, we consider that this acclimation may be involved in chloroplast ion transport caused by soil salinity induced osmotic stress. The silencing of the *TPK3* gene, which encodes the two-pore potassium (K+) channel protein, reduced the level of pmf in *Arabidopsis*, impairing the capacity for heat dissipation and causing reduced CO_2_ assimilation [31]. In addition, two groups describing the trans-thylakoid Cl^−^-flux protein bestrophin-like 1(Atbest1) in Arabidopsis, which was found to constitute a voltage-dependent chloride channel (AtVCCN1) involved in enhanced ΔpH and the fast activation of NPQ [32,33]. Thus, the ion transportome of chloroplasts appears to manage the pmf necessary to convert photochemical energy into photoprotection or other physiological functions.

To identify the master regulator involved in cross-tolerance is the key to elucidate the molecular network and adaptation mechanism of plant survival in fluctuating or intersecting stress conditions. Sustained alterations in the levels of key signaling metabolites, transcription factors, and epigenetic changes represent potential mechanisms that may underlie the stress imprint effect, which further facilitate rapid and more potent responses to subsequent attacks [34,35,36]. Although the present study does not touch upon signaling pathways and the molecular mechanisms, the photosynthetic performance of soil salinity-induced cross-tolerance to the subsequent drought conditions was comprehensively analyzed [37,38]. Our results clearly indicated that soil salinity pretreatment-induced photosynthetic acclimation could alleviate the damage induced by subsequent moderate drought conditions. These findings lay a foundation for further mechanistic studies of acclimation-induced cross-tolerance. Additional research is needed to establish the molecular mechanism of cross-tolerance, especially the regulatory networks of ROS, ABA, and the epigenetic regulation for photosynthesis in plants exposed to multiple stress conditions.

## 4. Materials and Methods

### 4.1. Plant Materials and Treatments

The tomato (*Solanum lycopersicum* L.) variety “Liao Yuan Duo Li” was used as experimental material; seeds were germinated in 50-hole seedling trays and transferred to plastic pots (13 cm × 13 cm) at the two-leaf stage. We conducted soil salinity pre-treatment and the subsequent drought treatment at the six-leaf stage. The experiments were conducted in the fully automatic controlled glass climate chamber at Shenyang Agricultural University from April to July in 2018. The temperature was controlled at 25/15 °C (day/night, 12 h/12 h), the humidity was approximately 50% during the day and 80% at night, and the plants were exposed to natural solar radiation, the light intensity was approximately 800 μmol·photons·m^−2^·s^−1^ at noon. Each seedling was watered about 50 mL per day to maintain a suitable water requirement during growth before the experiment. The pre-treatments were as follows: irrigation with 100 mL water (H_2_O100), 100 mL of 100 mM NaCl solution (NaCl100), 50 mL water (H_2_O50), and 50 mL of 100 mM NaCl solution (NaCl50) every morning for five days, followed by no water application for five days as the drought treatment. The measurements were conducted at the first (T1) and fifth (T5) day following soil salt pre-treatment, and the fifth day after the subsequent drought treatment (D5).

### 4.2. Measurement and Calculation of Leaf Gas Exchange Parameters

The GFS-3000 and DUAL-PAM-100 synchronous measuring instrument (Heinz Walz, Effeltrich, Germany) controlled by GFS-Win and Dual PAM v1.19 was used to analyze photosynthetic performance in vivo. The standard measurement procedures were conducted with minor modifications as we previously reported [39,40]. All measurements used the fourth leaf from the top of each plant; the area of the standard measuring head used was 1.3 cm^2^ with atmospheric CO_2_ concentrations (approximately 500 ppm) and temperature (approximately 22 °C), and the light intensity was 1100 μmol·photons·m^−2^·s^−1^. Gas exchange parameters, including net photosynthetic rate [Pn], intercellular CO_2_ concentration [Ci], stomatal conductance [gs], transpiration rate [E], water use efficiency [WUE], and stomatal limit value [Ls], were recorded when photosynthesis reached a steady state.

### 4.3. Measurement of the Fast Induction Curve of Chlorophyll a Fluorescence and Redox Kinetics of P700

A saturation pulse (300 ms, 10,000 μmol·photons·m^−2^·s^−1^) was applied using the automated routines provided by the Dual-PAM software to determine the fast induction curve of chlorophyll a fluorescence following tomato plant dark-adaptation for at least 30 min [39]. The activity of the donor and acceptor sides of PSII was reflected by a log timescale assessment of the relative fluorescence signal. The redox state of P700 was determined in vivo by using automated routines provided by the Dual-PAM software. Single turnover flash (ST, 50 ms) induction of the oxidation of PQ pools and multiple turnover flash (MT, 50 ms) induction of the full reduction of PQ pools in the presence of far-red light were used to measure the redox kinetics of P700. Balancing and calibration of dual-beam 870–830 nm signal difference was performed prior to each measurement. The complementary areas of ST and MT excitation signal change were used to calculate the functional pool sizes of PQ as follows: PQ size = MT − areas/ST − areas [39,40].

### 4.4. Simultaneous Measurement of Energy Conversion and Electron Transfer in PS(I) and PS(II)

The standard slow induction curve of chlorophyll fluorescence was recorded for 300 s to achieve the steady state of the photosynthetic apparatus following plant dark-adaptation for 30 min. A low intensity measuring light was used to detect the minimum fluorescence, F0; a saturating pulse (10,000 μmol·photons·m^−2^·s^−1^) was then applied to detect the maximal fluorescence, Fm; and application of a saturation pulse after illumination with far-red light for 10 s was used to measure the maximal change of the P700 signal, Pm. A saturating pulse (300 ms, 10,000 μmol·photons·m^−2^·s^−1^) was applied every 20 s after the actinic light (191 μmol·photons·m^−2^·s^−1^) was turned on to determine the maximum fluorescence signal (Fm′) and maximum P700^+^ signal (Pm′) following light adaptation. Rapid light response curves (RLCs) were generated using the previous achieved F0, Fm, and Pm with the standard measurement program of the Dual-PAM-100 software immediately after the finish of slow induction curve measurement. The light intensity of the RLC was changed every 30 s in the increasing sequence 29, 37, 55, 113, 191, 213, 349, 520, 778, 1197, and 1474 μmol·photons·m^−2^·s^−1^, and a saturating pulse was used to measure Fm′ and Pm′ after each period of actinic light [39,40]. The parameters used in this study were as follows: maximum photochemical quantum yield of PSII, Fv/Fm = (Fm − Fo) / Fm; effective quantum yield of PSII, Y(II) = (Fm’ − F) / Fm’; quantum yield of non-regulatory energy dissipation, Y(NO) = F / Fm; quantum yield of regulatory energy dissipation, Y(NPQ) = F / Fm′ − F / Fm; non-photochemical quenching in PSII, NPQ = (Fm – Fm’) / Fm′; photochemistry quantum yield of PSI photochemistry, Y(I) = (Pm′ − P) / Pm; quantum yield of non-photochemical energy dissipation owing to acceptor side limitation, Y(NA) = (Pm – Pm’) / Pm; quantum yield of PSI non-photochemical energy dissipation owing to donor side limitation, Y(ND) = (P − P0) / Pm; electron transfer rate of PSI, ETR(I) = Y(I) × PAR × 0.84 × 0.5; electron transfer rate of PSII, ETR(II) = Y(II) × PAR × 0.84 × 0.5; and estimated cyclic electron flow value (CEF), ETR(I) – ETR(II), where F is the light-adapted steady-state fluorescence, P0 is the zero P700 signal level for fully reduced P700, and P is the intermediate P700 signal prior to a saturation pulse in the presence of actinic light [41]. A transient increase in chlorophyll fluorescence was recorded after switching off actinic light to estimate the cyclic electron transport as previously described [8,42]. Plants were dark adapted at least 30 min and the actinic light intensity was set as 196 μmol·photons·m^−2^·s^−1^.

### 4.5. Electrochromic Shift (ECS) Signal Analysis

The ECS signal was measured by using the P515/535 module of the Dual-PAM-100. The measurement was conducted following plant dark-adaption for > 1 h with the balancing and calibration of the dual-beam 550 to 515 nm difference signal performed for each measurement; subsequently, the actinic light (630 µmol·m^−2^·s^−1^) was turned on for 300 s. The slow light-off-induced change of ECS signals reflected the total proton motive force (pmf) and its composition, ΔpH and ΔΨ. The time constant of the first order ECS relaxation (τECS) is inversely proportional to the proton conductivity of the thylakoid membrane through ATP synthase (gH^+^). Therefore, we estimated gH^+^ as the inverse of the decay time constant [1/τECS] [43,44]. The changes of ECS signal induced by ST were recorded following 1 h of dark adaptation to evaluate the integrity of the thylakoid membrane. Then, the same ST-induced ECS signal was recoded after the measured leaf was illuminated at 630 μmol·photons·m^−2^·s^−1^ for 6 min followed by 4 min dark adaptation to evaluate the activity of ATP-synthase [39].

### 4.6. Statistical Analysis

Statistical analyses were performed using SPSS version 22 (SPSS, Armonk, NY, USA). The Student’s t-test was used to generate every *p*-value. Means were considered to be significantly different at *p* < 0.05(*). The results were displayed as mean values of at least three independent biological replicates, standard deviation (SD) was calculated using the n values for each experiment. The measured plants were randomly selected from different treatments. All calculations were performed using MS Excel 2016 (Redmond, WA, USA) and the figures were generated using Origin version 12.0 (Systat, San Jose, CA, USA).

## 5. Conclusions

The soil salinity treatment and subsequent drought stress were conducted to clarify the cross-tolerance mechanism of tomato plants. Based on the results of this study, we conclude that the photosynthetic response mechanism of soil salinity-induced drought resistance of tomato plants occurs mainly by enhancing the photochemical reactions, inducing sustained NPQ, and maintaining high levels of cyclic electron flow and proton motive force (Figure 6). As the ion transportome of chloroplasts is capable of managing the pmf necessary to convert photochemical energy into photoprotection, we speculate that the soil salinity treatment causes ion imbalance in the chloroplast and thylakoid lumen in tomato leaves owing to the uptake of excessive sodium (Na^+^) and chloride (Cl^−^), allowing tradeoff between photoprotection and photochemistry reactions, and the achieved photosynthetic acclimation plays an important role in inducing cross-tolerance toward subsequent drought conditions. Future studies will benefit from further optimization of experimental design and measurement of evaluation indicators and the physiological and molecular characterizations—such as identifying the signaling pathways involved in the specific cross-tolerance between soil salinity and drought conditions are necessary. Nevertheless, the current study revealed that this cross-tolerance phenomenon offers numerous insights regarding photosynthetic acclimation in addition to soil water management during cultivation. In addition, this study has general implications for other similar crops especially vegetables because photosynthetic adaptation is relatively conserved across species.
